# Gold-silicon nanofiber synthesized by femtosecond laser radiation for enhanced light absorptance

**DOI:** 10.1186/1556-276X-9-255

**Published:** 2014-05-23

**Authors:** Abdul Salam Mahmood, Krishnan Venkatakrishnan, Bo Tan

**Affiliations:** 1 Department of Mechanical and Industrial Engineering, Ryerson University, 350 Victoria Street, Toronto, ON M5B 2K3, Canada; 2 Department of Aerospace Engineering, Ryerson University, 350 Victoria Street, Toronto, ON M5B 2K3, Canada

**Keywords:** Laser material processing, Nanomaterials, Photovoltaic

## Abstract

In this study, we devised a new concept for the precise nanofabrication of Au-Si fibrous nanostructures using megahertz femtosecond laser irradiation in air and atmospheric pressure conditions. The weblike fibrous nanostructures of Au thin layer on silicon substrate, which are proposed for the application of solar cells, exhibit a specific improvement of the optical properties in visible wavelength. Varying numbers of laser interaction pulses were used to control the synthesis of the nanofibrous structures. Electron microscopy analysis revealed that the nanostructures are formed due to the aggregation of polycrystalline nanoparticles of the respective constituent materials with diameters varying between 30 and 90 nm. Measurement of the reflectance through a spectroradiometer showed that the coupling of incident electromagnetic irradiation was greatly improved over the broadband wavelength range. Lower reflectance intensity was obtained with a higher number of laser pulses due to the bulk of gold nanoparticles being agglomerated by the mechanism of fusion. This forms interweaving fibrous nanostructures which reveal a certain degree of assembly.

**PACS:**

81.05.Zx; 81.07.-b

## Background

Manufacturing solar cells with an easy processing and inexpensive material has become the most important challenge for the future. Several articles focused on the enhancement of the spectral absorbance by modification of materials, improvement in electron-hole transport [1], and the usage of alternative wide-band-gap semiconductor materials [2]. Nanostructured material-based solar cells have attracted interest due to their characteristics and processing benefits. Silicon and metal nanowires, nanotubes, and nanorods which enable solar cells in decoupling light absorption from the direction of carrier transport have been studied by many researchers [3-6]. Minsung and Koichi demonstrated tin-catalyzed silicon nanowire solar cells fabricated by the hydrogen radical-assisted deposition method on a C-Si wafer, while Baxter and Aydil employed ZnO as a wide-band-gap semiconductor to construct dye-sensitized solar cells which exhibited an energy conversion efficiency of 0.5% with an internal quantum efficiency of 70%. Also, Huynh et al. studied polymer matrix solar cells using CdSe nanorods, achieving an efficiency of 1.7% [5]. The benefit of nanowires, nanotubes, and nanorods is the improvement of current densities because the diffusion length of minority carriers is much shorter than the thickness of the material required for optimal light absorption [7]. The application of nanofibrous structures in solar cells is the most promising method among other alternative approaches. Due to the high optical properties of nanoparticles, further research is also being carried out on nanoparticle-based dye-sensitized solar cells (DSSCs) [8-10].

Basically, metal nanoparticles exhibit remarkable properties departing from the bulk material counterparts due to their large surface area to volume ratio, high surface energy, and spatial confinement. For instance, gold nanoparticles exhibit a strong absorption peak near the 520-nm wavelength which cannot be observed in the bulk material due to surface plasmon oscillation modes of the conduction electrons in the nanoparticles [11]. Properties such as quantum confinement, surface plasmon resonance, enhanced catalytic activity, and superparamagnetism, among others, have been observed in nanomaterials to be varied as gold nanoparticle [7]. Laser scribing, laser patterning [12], and laser-induced ablation from a solid target are known as an alternative physical method for nanofabrication. Compaan et al. employed laser to scribe grooves of very narrow widths and superior profiles onto thin-film PV [13]. Rajeev et al. tried to increase metal absorption using a four-beam interference pattern creating hole-array structures to the surface [8]. Nakayama et al. investigated the effects of plasmon scattering on absorption and photocurrent collection in prototype GaAs solar cells decorated with size-controlled Ag nanoparticles by masked deposition through anodic aluminum oxide (AAO) templates and examined the size effects of hemispherical metal nanoparticle arrays [9]. Kume also investigated light emission from surface plasmon polaritons (SPPs) mediated by a metallic nanoparticle system consisting of Ag nanoparticles placed very close to an Al surface and prepared by depositing an Ag film on an Al film [10]. Novotný et al. [14] investigated the effect of the impact of a UV laser beam on thermally evaporated black gold and gold thin films with respect to their optical and structural properties. They observed that the absorptivity of the black gold film decreased with an increase in the number of laser pulses. The most recent effort includes using plasmonic metal nanoparticles to improve the efficiency of quantum dot solar cells and thin film solar cells [15,16].

The main difference between our nanofiber and other nanowire, nanotube, and nanorod structures in solar cell application is the ‘weblike and well-organized morphology structure’. Nanowire, nanotube, and nanorod morphology provides direct conduction paths for electrons from the point of injection to the collection electrode and allows for the decoupling of light absorption from the direction of carrier transport along the longitudinal direction only, while the weblike and network structure of nanofibers has inherent anisotropy with a large variety of morphology. Moreover, the dense network of nanofibers can provide a greater surface area of around 10^4^ times that that of untreated surfaces. In the present study, a femtosecond laser has been used to generate a nanofibrous structure on gold-silicon wafer. Different numbers of laser cycles were used to synthesize the nanofibrous structure with various dwell times. A spectroradiometer was used to measure reflectance to investigate the coupling of incident electromagnetic irradiation over the broadband wavelength range. The new structure revealed a higher reduction in visible light reflection when compared to an unstructured gold-silicon substrate.

## Methods

A thin gold film of 200-nm thickness was initially deposited onto a 0.02-Ω cm p-type silicon (100) wafer using an evaporator (e-beam) in the AMPEL Nanofabrication laboratory at the University of British Columbia (UBC). Four sets of these gold-silicon samples of 10 mm × 10 mm size were precisely cut using a dice saw and used for the present experiment. In order to obtain a large number of nanoparticles for analysis without damaging the surface of the target, laser cycles were gradually increased (2, 3, 4, and 5 cycles). The laser source is an all-diode-pumped, direct-diode-pumped Yb-doped fiber oscillator/amplifier system capable of producing variable pulse energies up to 10 mJ with a pulse frequency range between 200 kHz and 25 MHz. Average power varies between 0 and 20 W. In order to ablate the target material and create nanoparticles, the laser beam scanned the surface of the gold-sputtered silicon wafer in a 40 × 40 dot-array pattern. The laser beam dwell time at each dot point can be set at 0.5, 0.75, or 1.0 ms. The laser-irradiated samples were then characterized by scanning electrical microscopy (SEM), transmission electron microscopy (TEM), and energy-dispersive X-ray (EDX) analyses. A spectrophotometer (Ocean Optics, Dunedin, FL, USA) was used to measure the reflectance of the laser-irradiated samples by illumination with a wavelength in the range of 200 to 2,200 nm.

## Results and discussion

### Characterization of nanoparticle aggregation

**Figure 1:**
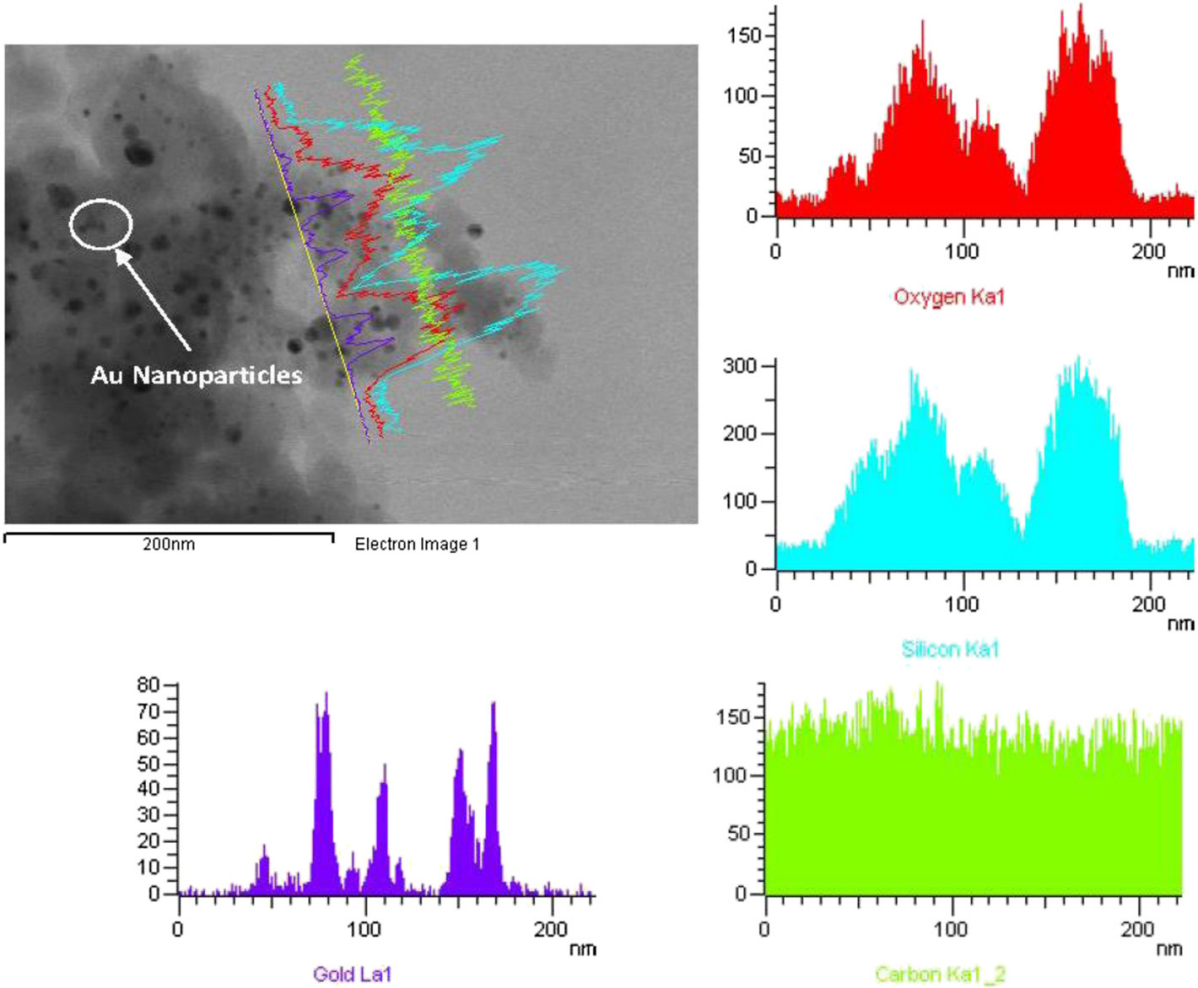
**TEM and EDX analyses.** TEM and EDX analyses show that a dense cloud of gold atoms (plume) firstly assembled in different laser spots of the gold target.

Figure 1 shows a TEM image of a gold-silicon nanofiber, accompanied with EDX analysis results. The figure shows that nanofibers consist of agglomerated silicon oxide nanoparticles with individual gold nanoparticles or a small cluster of gold nanoparticles dispersed in the cloud of silicon oxide nanoparticle agglomerates. It is also evident from the image that the diameter of gold particles is a fraction of that of silicon oxide particles.

The basic mechanism of femtosecond laser synthesis of nanoparticles could be explained in terms of the dynamic formation mechanism postulated by Sivakumar et al. [17] and Tan and Venkatakrishnan [18]. In brief, a dense cloud of atoms (plume) accumulated around the laser spot of the gold target during the course of ablation. This core was made up of a number of small gold atoms aggregated randomly due to the density fluctuation to form embryonic nanoparticles. Even when the ablation process had been terminated, at the end of the cycle, the aggregation continued, *per se* at a significantly slower growth rate with every new cycle until all atoms in the vicinity of the embryonic nanoparticles were depleted.

The significant difference in the melting/evaporation point of gold and silicon can be used to explain the large difference in particle size [19]. The evaporation/melting point of gold is much higher than that of silicon. As the cloud of plasma cools, the temperature of gold aggregation reduces to its melting point and particles solidify far before silicon particles reach the melting point. Therefore, silicon particles have much longer time to grow, leading to a much larger size.

**Figure 2:**
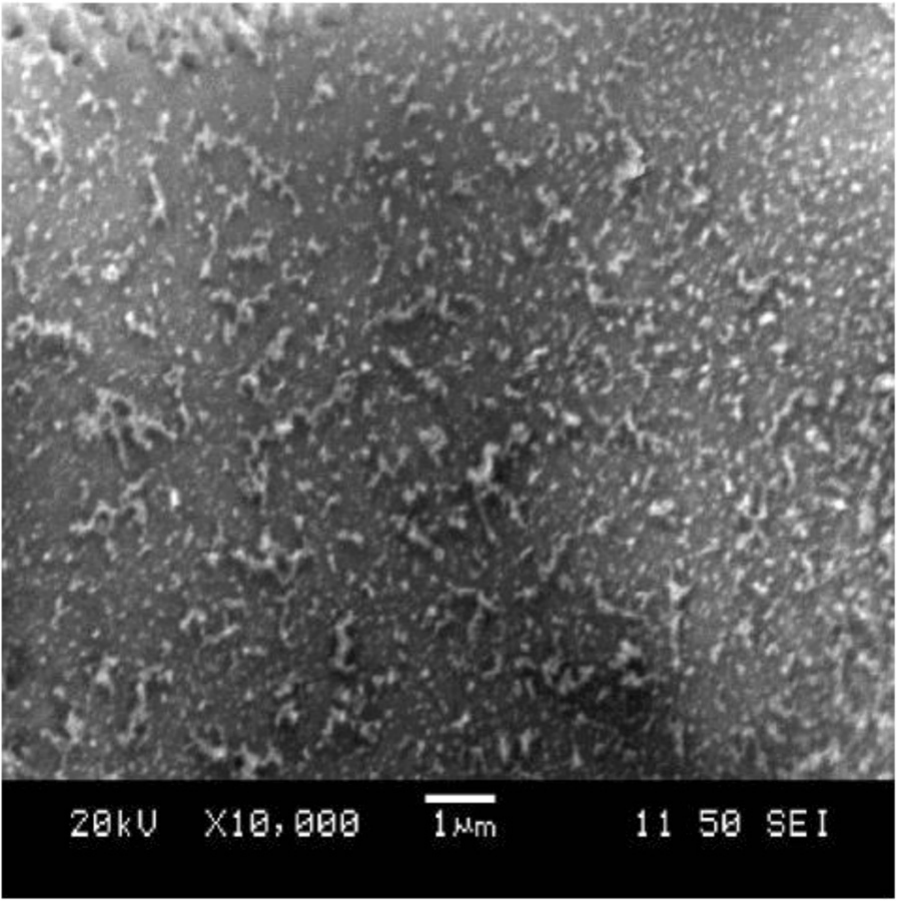
SEM image of a gold-silicon substrate irradiated with low cycles.

**Figure 3:**
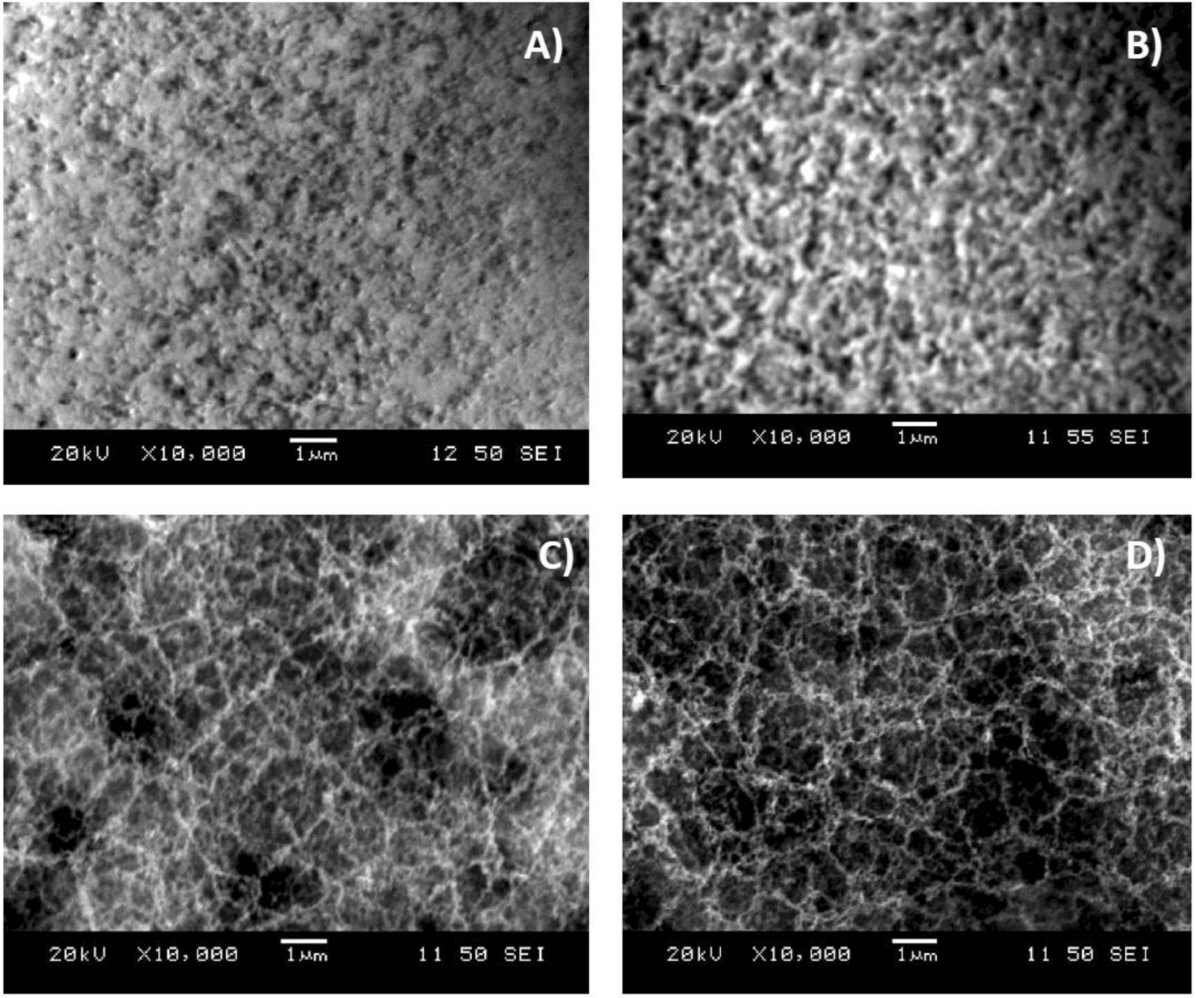
**SEM images of morphology transition with different cycles. (A)** Less than 2 cycles, **(B)** up to 2 cycles, **(C)** 4 cycles, and **(D)** 5 cycles.

The laser system used for this work has megahertz pulse frequency, so the energy of each laser pulse is in the order of nanojoules. It will generally need several pulses to create a dense plasma with a temperature high enough to evaporate both gold and silicon. Because of the large difference in evaporation points of gold and silicon, it is reasonable to speculate that gold and Si nanoparticles are initiated at different times, with silicon particles appearing first, at lower laser scanning cycles, and at a shorter dwell time.The formation of gold-silicon aggregated nanoparticles was observed starting at the second laser beam scanning cycle. Figure 2 shows nanofibers generated at a single laser beam scanning. With a single scanning cycle, short fibers mixed with large molten droplets were observed. The formation of fibrous aggregated nanoparticles was not evident.As the number of scanning cycles increases, the amount of molten droplets reduces and the aggregates grow longer, finally forming unique and uniform fibrous structures. Figure 3D shows typical weblike fibrous nanostructures formed due to the agglomeration of the bulk quantity of nanoparticles created during laser ablation at 5 cycles and 0.75-ms dwell time. Moreover, the fibrous nanostructures have relatively uniform diameters (around 50 nm) and do not have a wide range of variation in size distribution. In particular, the nanoparticles merge to form smooth chains.

**Figure 4:**
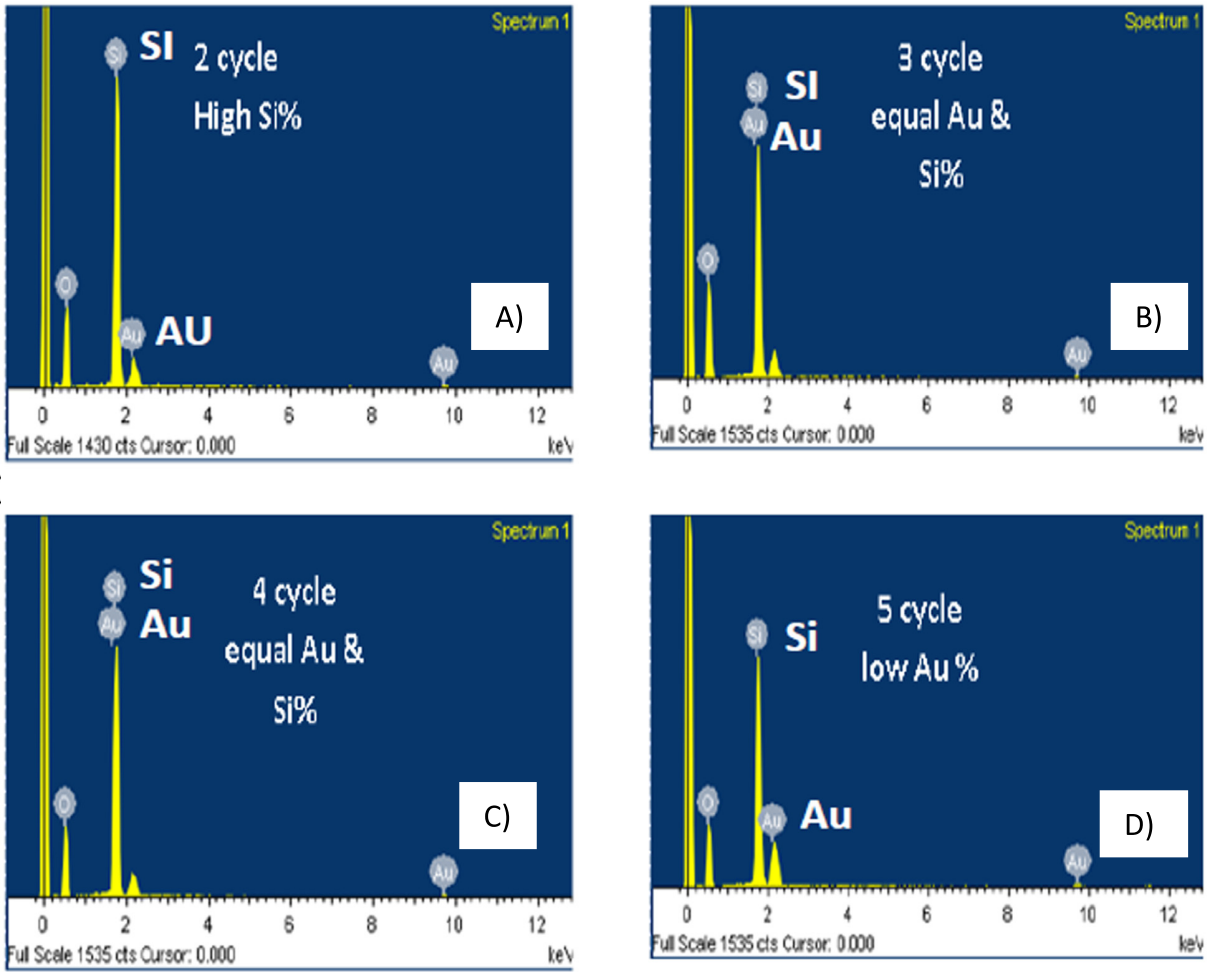
**EDX test showing the Au-Si percentage within different laser cycling. (A)** 2 cycles. **(B)** 3 cycles. **(C)** 4 cycles. **(D)** 5 cycles.

**Figure 5:**
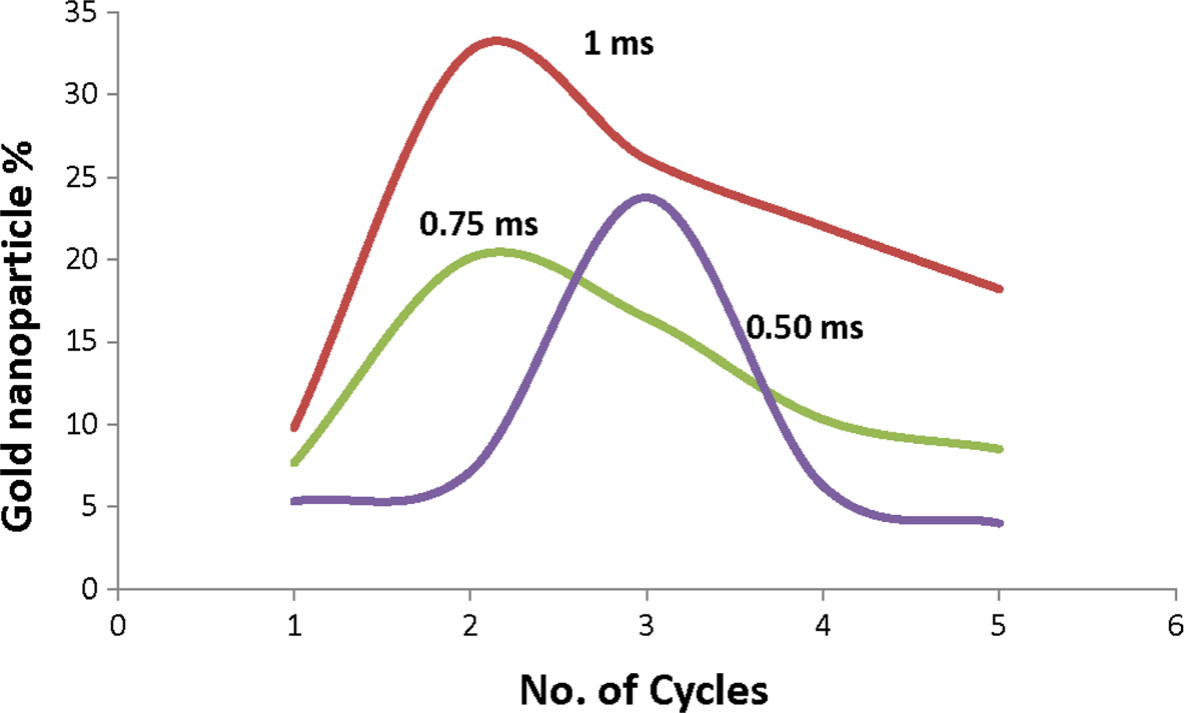
**Gold nanoparticle variation with number of cycles and dwell time.** 1 ms (red), 0.75 ms (green), and 0.50 ms (purple).

The most interesting phenomenon we observed is that the growth of silicon fibrous nanostructure begins first, followed by gold nanoparticle formation, until an equal quantity of these nanoparticles (approximately 50% of Si and Au) is formed at the third and fourth cycles. After that, the gold nanoparticle content drops. The gold content was measured by EDX analysis, as shown in Figure 4. Figure 5 shows the gold content at various laser machining parameters. The percentage of gold is obtained from EDX analysis results in Figure 4. Figure 5 shows that the gold content increases with the increase of laser beam dwell time. However, there is an optimum number of machining cycles at which the gold content reaches the highest. The reduction of gold content to a higher number of machining cycle may be due to the removal of the entire gold thin film [16] and the subsequent penetration of the laser beam to the Si substrate. As the gold thin film was completely consumed, the generated nanoparticle agglomerates entirely consist of silicon. Therefore, the overall detected gold content reduces.

### Light reflectance

**Figure 6:**
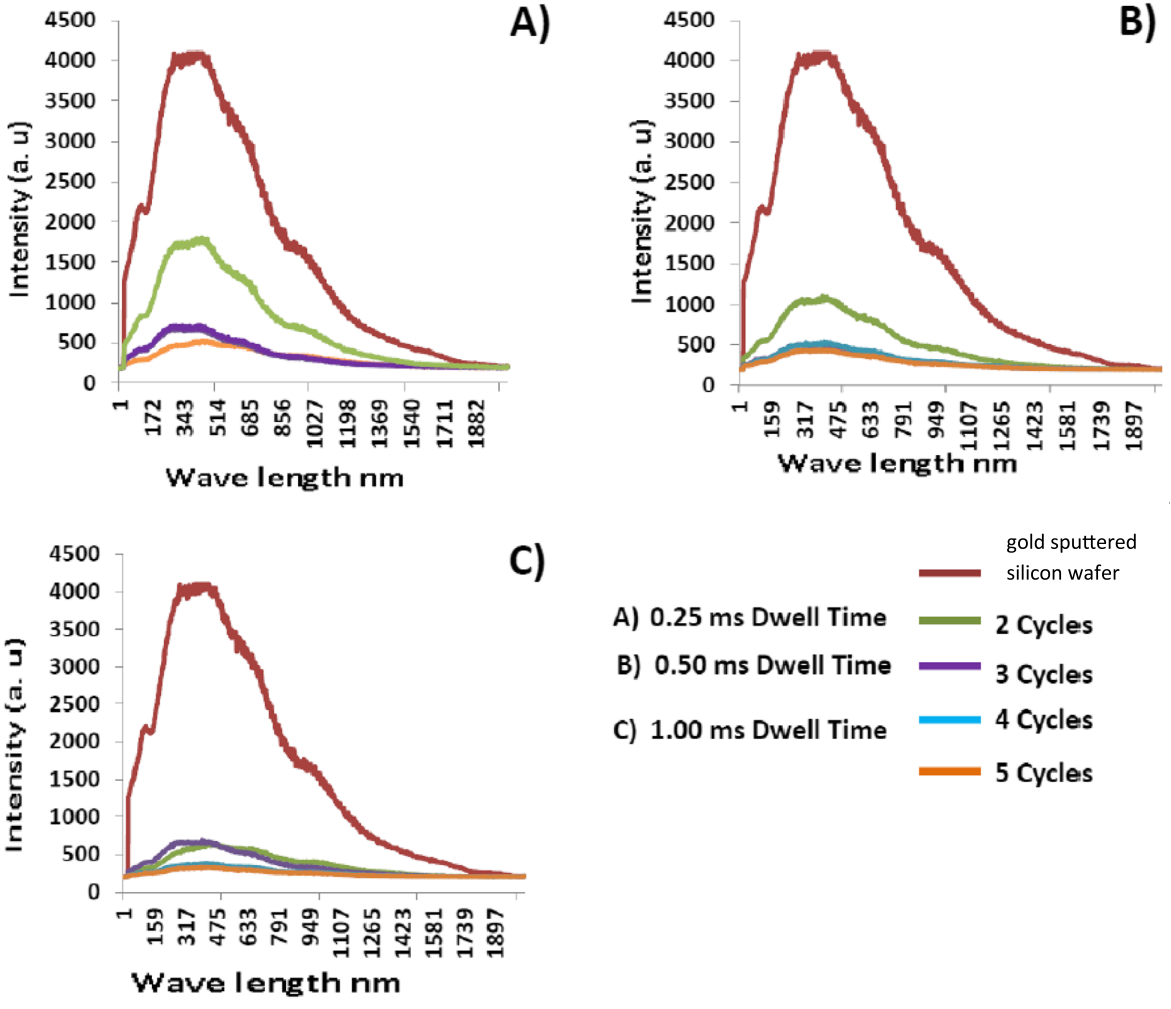
**Measured integrating reflectance spectra. (A)** 0.25 ms, (**B)** 0.50 ms, and **(C)** 1.00 ms.

The nanofibrous structure can significantly influence optical properties, which can differ considerably with those of the bulk materials. This type of structure enhances optical absorption due to surface plasmon excitation in the metal nanoparticle [10]. The micro-nanoscale surface roughness of the treated substrate could also increase light absorption due to the multiple reflections in micro-cavities and the variation of light incident angles. Metal surfaces with roughness on the scale of the optical wavelength are found to have a strong coupling of the incident light and become discolored as a result of selective surface plasmon absorption.In order to investigate the samples' enhanced absorption behavior in the visible region, a spectroradiometer was employed with a broad wavelength range of 250 to 1,200 nm. The measured integrating reflectance spectra are illustrated in Figure 6, where the red curve represents the reflectance of the unirradiated gold-silicon sample showing a high reflective intensity around 4,000 a.u.

The dark red curve represents the untreated sample, while the olive green, purple, light blue, and orange curves represent the reflection spectrum of the fibrous nanostructure layer with 2, 3, 4, and 5 cycles over visible wavelength, respectively, at different dwell times. The fibrous nanostructure increases the surface area by more than an order of magnitude which causes the radiation to pass through a longer distance before being reflected back. Therefore, a photon incident on a structured surface is likely to undergo more than one reflection before leaving the surface. Comparing the reflection spectrum to that of pure silicon nanofibers obtained from a previous experiment repeated on silicon wafer [20], we can conclude that the fibrous structure is the main attribute for light enhancement. The embedded gold particles will further enhance such multi-reflection, by increasing the intensity of reflection. This is evident from Figure 6A. At 2 scanning cycles and 0.25 ms of dwell time, the quantity of nanofiber is the lowest, but the percentage content of gold reaches the highest. Therefore, the enhancement effect is the most noticeable. It was observed that the reflectance decreased as the scanning cycle increased. As the scanning cycle increased, more fibrous nanostructures were generated and the thickness of the deposition increased, hence more effective in reflecting illumination. As the gold content reaches the highest at 2 or 3 cycles, we expect that the reflectance will drop to the lowest at these parameters. However, the results show that the reflectance reduces consistently with the increase of the number of cycles. This is attributed to the enhanced light absorptance of nanostructured silicon [20]. At higher number of cycles, the gold content reduces; however, the total quantity of the nanofiber increases. Therefore, the overall light absorptance of the treated substrate improves as the number of cycles increases. From these results, we can conclude that gold nanoparticles moderately enhance the light absorptance of silicon nanofiber. The enhancement is more effective when the quantity of silicon nanofibers is relatively low. If the deposition thickness of nanofiber is limited, embedding gold nanoparticles can be a method for enhancing light absorptance.

Moreover, the spectra exhibit a characteristic lower peak with the tail portion of the broadband extending towards the UV wavelength range. The width of the 519-nm peak is broadened and the height is lowered to a greater extent by introducing more laser shots. This spectral change indicates that the diameters of the nanoparticles are reduced more under irradiation of the laser with higher dwell time and more laser shots [20]. Moreover, when nanoparticles are sufficiently close together, interaction between neighboring particles arises. In simple words, when the longer dwell time creates a greater quantity of unique and homogenous distribution of the nanofibrous structures, the dipole created by the electric field of light induces a surface polarization charge, which effectively acts as a restoring force for free electrons.

## Conclusions

In summary, a simple and inexpensive method was implemented for synthesizing metal-semiconductor nanofibrous structures by using femtosecond laser material processing. The gold-silicon content ratio can be controlled by the number of interactive laser pulses. The highly improved coupling efficiency between light and the bulk quantity of gold nanoparticles may be attributed to the excitation of confined plasmon modes on the structured metal surfaces. These Au-Si solar cell nanofibrous structures may be a promising candidate for future photovoltaic application.

## Competing interests

The authors declare that they have no competing interests.

## Authors' contributions

At the time of this work, ASM was a Ph.D. candidate at Ryerson University. He conducted the experiment, developed the theory, and drafted the manuscript. KV was ASM's supervisor. He conceived the idea of this research work and planned the experiment. BT was ASM's co-supervisor. She contributed to the interpretation of experimental results and the theory development. She also revised the manuscript. All authors read and approved the final manuscript.

## References

[B1] Gebeyehu D Brabec CJ Sariciftci NS Solid-state organic/inorganic hybrid solar cells based on conjugated polymers and dye-sensitized TiO_2_ electrodes Thin Solid Films 2002 9 271 274

[B2] Keis K Magnusson E Lindstrom H Lindquist SE Hagfeldt A A 5% efficient photoelectrochemical solar cell based on nanostructured ZnO electrodes Sol Energy Mater Sol Cells 2002 9 51 58 10.1016/S0927-0248(01)00110-6

[B3] Minsung J Koichi K Synthesis and characterization of silicon nanowire using tin catalyst for solar cells application Mater Lett 2009 9 777 779 10.1016/j.matlet.2009.01.001

[B4] Baxter JB Aydil ES Nanowire-based dye-sensitized solar cells Appl Phys Lett 2005 9 053114 10.1063/1.1861510

[B5] Huynh WU Dittmer JJ Alivisatos AP Hybrid nanorod-polymer solar cells J Sci 2002 9 2425 10.1126/science.1069156 11923531

[B6] Grätzel M Photoelectrochemical cells Nature 2001 9 338 344 10.1038/35104607 11713540

[B7] Perez M Iron oxide nanoparticles: hidden talent Nat Nanotechnol 2007 9 9 535 536 10.1038/nnano.2007.282 18654361

[B8] Rajeev P Bagchi A Kumar G Nanostructures, local fields, and enhanced absorption in intense light–matter interaction Optic Letter 2004 9 22 2662 2664 10.1364/OL.29.002662 15552678

[B9] Nakayama K Tanabe K Atwater H Plasmonic nanoparticle enhanced light absorption in GaAs solar cells Appl Phys Lett 2008 9 121904 10.1063/1.2988288

[B10] Kume T Hayashi S Yamamoto K Light emission from surface plasmon polaritons mediated by metallic fine particles Phys Rev B 1997 9 7 4774 4782 10.1103/PhysRevB.55.4774

[B11] Seung H Choi K David J Grigoropoulos CP Nanosecond laser ablation of gold nanoparticle films Appl Phys Lett 2006 9 141126 10.1063/1.2360241

[B12] Westin P-O Zimmermann U Ruth M Edoff M Next generation interconnective laser patterning of CIGS thin film modules Sol Energy Mater Sol Cells 2011 9 4 1062 1068 10.1016/j.solmat.2010.11.015

[B13] Compaan AD Matulionis I Nakade S Optimization of Laser Scribing for Thin-Film PV Modules 1997 National Renewable Energy Laboratory: Golden

[B14] Novotný M Fitl P Sytchkova A Lancok A Pokorný P Najdek D Bocan J Pulsed laser treatment of gold and black gold thin films fabricated by thermal evaporation J Phys 2009 9 2 327 331

[B15] Hairen T Rudi S Smets AHM Miro Z Plasmonic light trapping in thin film silicon cells with improved self assembled silver nanoparticles Nano Lett 2012 9 8 4070 4076 10.1021/nl301521z 22738234

[B16] Jiang W Mangham SC Reddy VR Manasreh MO Weaver BD Surface plasmon enhanced intermediate band based quantum dots solar cell Sol Energy Mater Sol Cells 2012 9 44 49

[B17] Manickam S Venkatakrishnan K Tan B Venkataramanan V Study of silicon nanofibrous structure formed by laser irradiation in air Opt Express 2009 9 16 13869 13874 10.1364/OE.17.013869 19654793

[B18] Tan B Venkatakrishnan K Synthesis of fibrous nanoparticle aggregates by femtosecond laser ablation in air Opt Exp 2009 9 2 1064 1069 10.1364/OE.17.001064 19158924

[B19] Amirkianoosh K Palneet Singh W Venkatakrishnan K Tan B Synthesis of 3D nanostructured metal alloy of immiscible materials induced by megahertz repetition femtosecond laser pulses Nanoscale Res Lett 2012 9 1 518 10.1186/1556-276X-7-518 22999219 PMC3599459

[B20] Mahmood A Sivakumar M Venkatakrishnan K Tan B Enhancement in optical absorption of silicon fibrous nanostructure produced using femtosecond laser ablation Appl Phys Lett 2009 9 034107 10.1063/1.3168499

